# WNT activation by lithium abrogates *TP53* mutation associated radiation resistance in medulloblastoma

**DOI:** 10.1186/s40478-014-0174-y

**Published:** 2014-12-24

**Authors:** Nataliya Zhukova, Vijay Ramaswamy, Marc Remke, Dianna C Martin, Pedro Castelo-Branco, Cindy H Zhang, Michael Fraser, Ken Tse, Raymond Poon, David JH Shih, Berivan Baskin, Peter N Ray, Eric Bouffet, Peter Dirks, Andre O von Bueren, Elke Pfaff, Andrey Korshunov, David TW Jones, Paul A Northcott, Marcel Kool, Trevor J Pugh, Scott L Pomeroy, Yoon-Jae Cho, Torsten Pietsch, Marco Gessi, Stefan Rutkowski, Laszlo Bognár, Byung-Kyu Cho, Charles G Eberhart, Cecile Faure Conter, Maryam Fouladi, Pim J French, Wieslawa A Grajkowska, Nalin Gupta, Peter Hauser, Nada Jabado, Alexandre Vasiljevic, Shin Jung, Seung-Ki Kim, Almos Klekner, Toshihiro Kumabe, Boleslaw Lach, Jeffrey R Leonard, Linda M Liau, Luca Massimi, Ian F Pollack, Young Shin Ra, Joshua B Rubin, Erwin G Van Meir, Kyu-Chang Wang, William A Weiss, Karel Zitterbart, Robert G Bristow, Benjamin Alman, Cynthia E Hawkins, David Malkin, Steven C Clifford, Stefan M Pfister, Michael D Taylor, Uri Tabori

**Affiliations:** Division of Hematology/Oncology, The Hospital for Sick Children, Department of Pediatrics, University of Toronto, 555 University Avenue, M5G 1X8 Toronto, ON Canada; The Arthur and Sonia Labatt Brain Tumour Research Centre, The Hospital for Sick Children, Toronto, ON Canada; Division of Neurosurgery, The Hospital for Sick Children, Toronto, ON Canada; Department of Laboratory Medicine and Pathobiology, University of Toronto, Toronto, ON Canada; Division of Pathology, The Hospital for Sick Children, Toronto, ON Canada; Cellular Imaging Core, STTARR Innovation Facility/Radiation Medicine Program and Department of Applied Molecular Oncology, Ontario Cancer Institute/Princess Margaret Hospital, Toronto, ON Canada; Program in Developmental and Stem Cell Biology, The Hospital for Sick Children, Toronto, ON Canada; Institute of Medical Science, Faculty of Medicine, University of Toronto, Toronto, ON Canada; Division of Orthopedic Surgery, The Hospital for Sick Children, Toronto, ON Canada; Department of Surgery, Faculty of Medicine, University of Toronto, Toronto, ON Canada; Department of Paediatric Laboratory Medicine, The Hospital for Sick Children, Toronto, ON Canada; Department of Pediatrics and Adolescent Medicine, Division of Pediatric Hematology and Oncology University Medical Center, Gottingen, Germany; Division of Molecular Genetics, University Hospital, Heidelberg, Germany; Department of Paediatric Oncology, Haematology and Immunology, University Hospital, Heidelberg, Germany; CCU Neuropathology, DKFZ and Department of Neuropathology, University Hospital, Heidelberg, Germany; Princess Margaret Cancer Centre, University Health Network, Toronto, ON Canada; Department of Neurology, Boston Children’s Hospital, Harvard Medical School, Boston, MA USA; Department of Neurology, Stanford University, San Francisco, CA USA; Institute of Neuropathology, University of Bonn Medical Centre, Bonn, Germany; Department of Paediatric Haematology and Oncology, University Medical Center Hamburg-Eppendorf, Hamburg, Germany; Department of Neurosurgery, University of Debrecen, Clinical Centre, Debrecen, Hungary; Division of Pediatric Neurosurgery, Seoul National University Children’s Hospital, Seoul, South Korea; Departments of Pathology, Ophthalmology and Oncology, John Hopkins University School of Medicine, Baltimore, MD USA; Department of Paediatrics, Institute of Hematology and Pediatric Oncology, Lyon, France; Division of Oncology, Cincinnati Children’s Hospital Medical Center, Cincinnati, OH USA; Department of Pathology and Neurology, Erasmus Medical Center, Rotterdam, Netherlands; Department of Pathology, The Children’s Memorial Health Institute, Warsaw, Poland; Departments of Neurological Surgery, University of California San Francisco, San Francisco, CA USA; Department of Pediatrics, University of California San Francisco, San Francisco, CA USA; Department of Pediatrics, Semmelweis University, Budapest, Hungary; Department of Pediatrics, Division of Haemato-Oncology, McGill University, Montreal, QC Canada; Centre de Pathologie EST, Groupement Hospitalier EST, Hospices Civils de Lyon, Bron, France; Department of Neurosurgery, Université Chonnam National University Research Institute of Medical Sciences, Chonnam National University Hwasun Hospital and Medical School, Hwasun-gun, Chonnam South Korea; Department of Neurosurgery, Tohoku University Graduate School of Medicine, Sendai, Japan; Department of Pathology and Molecular Medicine, Division of Anatomical Pathology, McMaster University, Hamilton, ON Canada; Department of Neurosurgery, Division of Pediatric Neurosurgery, Washington University School of Medicine and St. Louis Children’s Hospital, St. Louis, MO USA; Department of Pediatrics, Anatomy and Neurobiology, Washington University School of Medicine and St. Louis Children’s Hospital, St. Louis, MO USA; Department of Neurosurgery, David Geffen School of Medicine at UCLA, Los Angeles, CA USA; Pediatric Neurosurgery, Catholic University Medical School, Rome, Italy; Department of Neurological Surgery, University of Pittsburgh School of Medicine, Pittsburgh, PA USA; Department of Neurosurgery, University of Ulsan, Asan Medical Center, Seoul, South Korea; Department of Neurosurgery, School of Medicine and Winship Cancer Institute, Emory University, Atlanta, GA USA; Department of Hematology & Medical Oncology, School of Medicine and Winship Cancer Institute, Emory University, Atlanta, GA USA; Department of Neurological Surgery, University of California San Francisco, San Francisco, CA USA; Department of Paediatrics, University of California San Francisco, San Francisco, CA USA; Department of Pediatric Oncology, University Hospital Brno and Masaryk University School of Medicine, Brno, Czech Republic and Regional Centre for Applied Molecular Oncology, Masaryk Memorial Cancer Institute, Brno, Czech Republic; Program in Genetics and Genome Biology, The Hospital for Sick Children, Toronto, ON Canada; Northern Institute for Cancer Research, Newcastle University, Newcastle-upon-Tyne, United Kingdom; Departamento de Ciências Biomédicas e Medicina, Regenerative Medicine Program, Universidade do Algarve, 8005-139 Faro, Portugal; Centre for Molecular and Structural Biomedicine, CBME/IBB, LA. University of Algarve, 8005-139 Faro, Portugal; Department of Medical Biophysics, University of Toronto, Toronto, ON Canada; Department of Immunology, Genetics and Pathology, Uppsala University, Rudbeck Laboratory, Uppsala, Sweden; Department of Clinical Genetics, Uppsala University Hospital, Uppsala, Sweden

## Abstract

*TP53* mutations confer subgroup specific poor survival for children with medulloblastoma. We hypothesized that WNT activation which is associated with improved survival for such children abrogates *TP53* related radioresistance and can be used to sensitize *TP53* mutant tumors for radiation. We examined the subgroup-specific role of *TP53* mutations in a cohort of 314 patients treated with radiation. *TP53* wild-type or mutant human medulloblastoma cell-lines and normal neural stem cells were used to test radioresistance of *TP53* mutations and the radiosensitizing effect of WNT activation on tumors and the developing brain. Children with WNT/*TP53* mutant medulloblastoma had higher 5-year survival than those with SHH/*TP53* mutant tumours (100% and 36.6% ± 8.7%, respectively (p < 0.001)). Introduction of *TP53* mutation into medulloblastoma cells induced radioresistance (survival fractions at 2Gy (SF2) of 89% ± 2% vs. 57.4% ± 1.8% (p < 0.01)). In contrast, β-catenin mutation sensitized *TP53* mutant cells to radiation (p < 0.05). Lithium, an activator of the WNT pathway, sensitized *TP53* mutant medulloblastoma to radiation (SF2 of 43.5% ± 1.5% in lithium treated cells vs. 56.6 ± 3% (p < 0.01)) accompanied by increased number of γH2AX foci. Normal neural stem cells were protected from lithium induced radiation damage (SF2 of 33% ± 8% for lithium treated cells vs. 27% ± 3% for untreated controls (p = 0.05). Poor survival of patients with *TP53* mutant medulloblastoma may be related to radiation resistance. Since constitutive activation of the WNT pathway by lithium sensitizes *TP53* mutant medulloblastoma cells and protect normal neural stem cells from radiation, this oral drug may represent an attractive novel therapy for high-risk medulloblastomas.

**Electronic supplementary material:**

The online version of this article (doi:10.1186/s40478-014-0174-y) contains supplementary material, which is available to authorized users.

## Introduction

Medulloblastoma is the most common malignant brain tumor of childhood [1]. Survival of these children has improved in the last two decades due to collaborative studies utilizing aggressive surgical resection, high dose craniospinal irradiation and chemotherapy [2-4]. This aggressive approach to therapy has come at a severe cost with the majority patients suffering significant long-term neurocognitive, endocrine and other toxicities [5]. Recent progress in the molecular stratification of medulloblastoma has revealed striking heterogeneity that has led to stratification into several genetically distinct clinical subgroups (WNT, SHH, Group 3, Group 4) which respond differently to current therapies [6].

Of the four subgroups, patients with WNT subgroup medulloblastoma exhibit an excellent overall survival (OS), those with SHH medulloblastoma have an intermediate long-term survival, whereas those with Groups 3 and 4 disease have particularly decimal outcomes [7,8]. Recent studies highlighted the role of additional genetic alterations in identifying risk factors in each molecular subgroup. For example, iso17q together with *MYC* amplification define the high risk patients in group 3, but not group 4. In contrast, Group 4 patients with whole chromosome 11 loss, or chromosome 17 gain together with loss of chromosome 10p, demonstrate better survival. Absence of *GLI2* amplification and 14q loss identify a low-risk patient population, chromothripsis is found in SHH patients with inferior outcomes, and monosomy of chromosome 6 carries favorable prognostic value only within the WNT subgroup [9].

Our group has recently uncovered the role of *TP53* mutations in clinically distinct subgroups of medulloblastoma [10]. *TP53* mutations are restricted to the SHH and WNT subgroups. In the former it is associated with poor survival while in the latter, this survival disadvantage is not seen [10].

*TP53* is mutated or deleted in over 50% of human cancers resulting in loss of p53-associated apoptosis, cell cycle arrest or DNA brake/repair response [11]; Loss of normal p53 function and resultant impaired G1 check point control correlates with increased resistance to both low- and high-dose ionizing radiation in several cancers including medulloblastoma [12-21]. It is argued that, though tissue specific, *TP53* mutation status can predict tumor radiosensitivity and a patient’s response to radiation therapy [19,20].

Craniospinal radiation therapy remains the cornerstone of treatment in childhood medulloblastoma. It is, therefore, reasonable to hypothesize that the poor survival of patients with *TP53* mutant medulloblastoma is related to radioresistance of these cancers. In contrast, in 10-15% of medulloblastoma displaying activation of the WNT/β-catenin pathway *TP53* mutant status does not negatively influence survival [18,22,23]. In the WNT subgroup, patients with either wild-type or mutant *TP53* tumors respond equally well to radiotherapy. Most WNT subgroup tumors harbor activating mutations in exon 3 of the β-catenin gene, and this genetic alteration is a universally favorable prognostic marker for both average- and high-risk patients, and long term patient survival exceeds 90% [3,6,7,22,24-28].

Lithium is a non-competitive and specific inhibitor of *GSK3*β which is a negative regulator of the WNT pathway. Lithium mimics canonical WNT activation marked by nuclear translocation of β-catenin and transcriptional activation of downstream targets [29-31]. Since lithium can be safely administered to patients and crosses the brain–blood barrier [32], it might constitute a therapeutic means to induce radiosensitivity and improve survival in SHH/mutant *TP53* patients.

To test our hypothesis we first assessed the impact of *TP53* mutations on survival of children with medulloblastoma treated with craniospinal irradiation. We then examined the role of lithium-mediated WNT activation in abrogating the radioresistance observed in *TP53* mutant medulloblastoma cells. Our results suggest that lithium administration with radiation therapy may be a safe and efficacious therapy for childhood medulloblastoma.

## Materials and Methods

Patient and sample description: We collected clinical and biological data on 314 patients from the Hospital for Sick Children (Toronto, Canada), Division of Molecular Genetics and Department of Paediatric Oncology, Haematology and Immunology, University Hospital (DKFZ) (Heidelberg, Germany), Northern Institute for Cancer Research, Newcastle University (Newcastle, United Kingdom) [33], Institute of Neuropathology, University of Bonn Medical Centre (Bonn, Germany) [34] and the Medulloblastoma Advanced Genomics International Consortium (MAGIC) [35] cohorts. Patients between 5 and 19 years of age were eligible for this study. We excluded patients younger the 5 years of age at diagnosis under the assumption that they were not eligible for radiation therapy according to the most of the current medulloblastoma protocols. All patient samples were procured in accordance with the Research Ethics Board of their corresponding institution. For 300 (95.5%) patients complete clinical and survival data were available for analysis. Samples from all centers were either obtained as frozen tissue or formalin fixed paraffin embedded (FFPE) biopsies and processed to determine subgroup β-catenin and *TP53* status as previously described [10].

Cell line work: Human medulloblastoma wild-type *TP53* cells lines: ONS76, D283MED, MEB-MED-8A and *TP53* mutant cell lines: UW228 and Daoy were cultured as previously described [36,37]. Normal neural stem cells (NNSC) Hf5205 were cultured as previously described [38]. ONS76, D283MED, MEB-MED-8A, UW228 and Daoy cells were provided by Taylor Lab (The Hospital for Sick Children, Toronto) and Brain Tumor Research Centre (BTRC) repository (The Hospital for Sick Children, Toronto). NNSC, Hf5205, were provided by Dirks Lab (The Hospital for Sick Children, Toronto). All cells were authenticated including verification of *TP53* status. Lithium chloride (Sigma, 02685-1EA), was used at a final concentration of 2 mM in regular culture medium. Radiation exposure was performed using a Nordion Gammacell 40 irradiator with the central dose rate 1.24 Gy/min. Single discrete doses of 1 to 8Gy were delivered according to experimental design.

Clonogenic experiments: To generate radiation sensitivity curves cells were seeded at specific numbers in triplicates into 10 cm dishes to yield between 50 and 150 colonies. Cells were allowed to adhere overnight and then irradiated with the range of single doses from 1 to 5Gy. The medium was changed after irradiation and cells were maintained at 37°C for 14 days to allow colony formation. The colonies (50 cells/colony threshold) were then fixed and stained with Crystal violet (0.1%) and counted manually. The surviving fractions were calculated after adjustment for plating efficiency of non-irradiated controls as follows [39]:

$$ \mathrm{S}\mathrm{F}\left( survival\  fraction\right)=\left[\left(\mathrm{number}\ \mathrm{of}\ \mathrm{colonies}\ \mathrm{observed}\right)\div \left(\mathrm{number}\ \mathrm{of}\ \mathrm{cells}\ \mathrm{plated}\right)\div \mathrm{P}\mathrm{E}\left( plating\  efficiency\  of\  control\right)\right] $$

$$ \mathrm{P}\mathrm{E}\left( plating\  efficiency\  of\  control\right)=\left(\mathrm{number}\ \mathrm{o}\mathrm{f}\ \mathrm{colonies}\ \mathrm{o}\mathrm{bserved}\ \mathrm{in}\ \mathrm{n}\mathrm{o}\mathrm{n}\hbox{-} \mathrm{irradiated}\ \mathrm{sample}\right)\div \left(\mathrm{number}\ \mathrm{o}\mathrm{f}\ \mathrm{cells}\ \mathrm{plated}\right) $$

Survival fraction at 2 Gray (SF2) calculations: To fit the clinical relevance, the radiation dose of 2Gy was selected to match the daily fraction size commonly used in paediatric clinical practice [40]. The SF2 was calculated as following:

$$ \mathrm{S}\mathrm{F}2=\left[\left(\mathrm{number}\ \mathrm{of}\ \mathrm{colonies}\ \mathrm{observed}\ \mathrm{after}\ \mathrm{treatment}\ \mathrm{with}\ 2\mathrm{Gy}\right)\div \left(\mathrm{number}\ \mathrm{of}\ \mathrm{cells}\ \mathrm{plated}\right)\div \mathrm{P}\mathrm{E}\left( plating\  efficiency\  of\  control\right)\right] $$

Sensitizer enhancement ratio (SER) calculations: SER was calculated as the radiation dose needed for radiation alone divided by the radiation dose together with of lithium needed to eliminate 90% of cells [36].

Same corresponding cell lines simultaneously maintained without lithium chloride were used as controls. Data from three independent experiments performed in triplicate were each plotted as a function of radiation dose on a semi-logarithmic scale.

Transfection experiments: For each cell line, 3 × 10^5^ cells were transiently transfected in parallel with 2 μg of the plasmid-of-interest DNA or 2 μg pcDNA3 control plasmid (either GFP- or FLAG-tagged) by nucleoporation, as per the manufacturer’s instructions, using the Amaxa Mouse Neural Stem Cell Kit (Nucleofector, Lonza, VPG-1004) and X001 program. At 24 hours post transfection the transfection efficiency of 59.9% ± 12.3% for the pcDNA3-TP53R175H/pcDNA3-GFP plasmid combination and 90% for the pcDNA3-S33Y/pcDNA3-FLAG plasmid combination was estimated by manual counting of GFP- and FLAG- positive cells under immunofluorescent microscopy. Subsequently, cells were irradiated between 24 and 48 hours and plated for clonogenic assay as described above. Plasmids used: pcDNA3-TP53R175H, pcDNA3 (courtesy of Dr. David Malkin, The Hospital for Sick Children, Toronto), and pcDNA3-S33Y β-catenin [41] obtained from Addgene: http://www.addgene.org/.

Clonogenic experiments with combination of lithium and radiation exposure: ONS76 and UW228 cells were cultured, as described above. Cells were exposed to 2 mM lithium chloride for 24 hours, and then irradiated with the range of doses from 2 to 8Gy. Drug-containing medium was replaced with normal growth medium. Clonogenic experiments were performed as described above.

Immunofluorescence – β-catenin nuclear translocation: Cells transfected with S33Y-CTNNB1 mutation and/or pre-treated with 2 mM of lithium chloride for 24 hours were cultured as described above. β-catenin expression was visualized by immunofluorescence using a Quorum Spinning Disk Confocal Microscope. The antibodies used were: a mouse anti-β-catenin (BD Transduction Labs, 6101153) and a donkey-anti-mouse IgG conjugated with Alexa Fluor 488 (Invitrogen, A21202); both were used at 1:500 dilution. All experiments were repeated three times.

β-catenin luciferase reporter assay: For the reporter assay, 1 × 105 cells were plated in 24-well plates in triplicate per sample and left to attach overnight. Cells were transiently transfected in parallel with either 0.5 μg of the TCF reporter construct (M50 Super 8xTOPFlash) DNA or 0.5 μg of the mutated reporter construct (M51 Super 8×FOPFlash) DNA [39,42] together with 1 ng of Renilla DNA. Plasmids were obtained from Addgene: http://www.addgene.org/. Transfections were performed using Lipofectamine 2000 (Invitrogen, 11668–027) according to manufacturer’s protocol. After 4 hr post transfection medium was replaced with the regular growth medium and cells were treated with 2 mM of lithium chloride for 24 hr. TCF-mediated transcriptional activity was determined by the ratio of TOPFlash/FOPFlash luciferase activity, normalized to the Renilla luciferase activity, using Promega Dual Luciferase Reporter Assay System, (Promega, E1960). 3 independent experiments were done.

Phospho-histone H2AX (γH2AX) foci immunofluorescent microscopy and imaging: Medulloblastoma and NNSC cells were cultured on cover slips in 6-well dishes and fixed as described above. Cells were pre-treated with 2 mM lithium chloride for 24 hours and then exposed to 5Gy single dose of radiation. Untreated and non-irradiated cells were used as control. Specimens were collected at 30 min, 4 and 24 hours post radiation. At appropriate time points, cells were fixed and processed as described above. The antibodies used were: a mouse anti-phospho-histone-H2AX (Ser139) (Millipore, 05–636), 1:800 dilution, and an Alexa Fluor 488 donkey-anti-mouse IgG (Invitrogen, A21202), 1:500 dilution. For quantification of γH2AX foci 3 independent experiments were performed. Between 100 and 250 nuclei were examined and foci were counted using Perkin Elmer Velocity 6.0.1 software (Perkin Elmer, USA) with 0.2um cut off for foci size. The following procedure was applied to decrease inter-experimental variability: 1) all 3 experiments were pooled together and individual values were used for analysis [43]; 2) average number of foci per nucleus was calculated; 3) outliers, which were estimated as nuclei containing > 2 standard deviation (2 SD) from the average for the treatment subgroup, were eliminated; 4) baseline number of foci per nucleus was estimated in the untreated sample as average plus 2 SD; 5) to remove background noise nuclei with baseline or less number of foci were eliminated from experimental samples and the remaining nuclei were considered γH2AX-positive for further calculations; 6) number of foci in γH2AX-positive nuclei was calculated and expressed as an average number of foci per nuclei; 7) comparison was done between treatments using average number of foci per nucleus and number of γH2AX-positive cells as defined above.

Normal neuronal stem cell viability experiments: NNSC (Hf5205) were cultured and pre-treated with lithium chloride as previously described. Cells were counted five days after radiation using a Cell Viability Analyzer (Vi-Cell XR, Beckman Coulter). Survival curves were generated as described above. The figure was plotted from three independent experiments performed in triplicate each.

Statistical analysis: Overall survival was estimated using the Kaplan-Meier method with significance (α = 0.05) based on long-rank test, using Stata 12 for OS X. OS was defined as the interval between the date of diagnosis and the date of death of any cause or the date of the last follow-up visit.

For biological data results were expressed as the mean ± SE of three or more independent experiments carried out in triplicate. Statistically significant differences between samples were determined using Student 2-tailed *t*-test and two-way ANOVA with the Bonferroni correction method, when appropriate, using GraphPad Prism version 5.00 for Windows, GraphPad Software, San Diego California USA, www.graphpad.com; p value < 0.05 was considered significant. The error bars in the figures represent SEs.

## Results

Impact of *TP53* mutations on children treated with irradiation. In order to assess the role of *TP53* mutations in medulloblastoma patients who received radiotherapy, we selected patients between 5 and 19 years old who received craniospinal radiation according to local standard of care protocols. The complete characteristic of the cohort is summarized in Table 1. We identified somatic *TP53* mutations in 16.9% (53/314) of cases. Median age at diagnosis and gender distribution was not different between wild-type or mutant *TP53* patients. We observed a higher proportion (p < 0.01) of metastatic disease in *TP53* wild-type patients (Table 1). Anaplastic histology was more prevalent in *TP53* mutant patients (p < 0.001).

**Table 1:** **Patient characteristics according to** ***TP53*** **status**

**Variable**	***TP53*** **wild-type**	***TP53*** **mutant**	**p-value**
N	261/314 (83.1%)	53/314 (16.9%)	
Age (median, range)	9.0 (5.0 – 18.5)	10.0 (5.0 – 17.0)	p = 0.4
Gender (male)	154/261 (59%)	25/53 (47.2%)	p = 0.1
Histology			
LCA	31/261 (11.9%)	23/53 (43.4%)	p = 0.0001*
Classic	204/261 (78.2%)	25/53 (47.1%)	
Desmoplastic/nodular	21/261 (8.0%)	2/53 (3.8%)	
Missing	5 /261 (1.9%)	3/53 (5.7%)	
M+ disease	80/261 (30.7%)	7/53 (13.2%)	p = 0.01
Subgroups			
WNT	72/261 (27.6%)	15/53 (28.3%)	p = 0.003**
SHH	59/261 (22.6%)	36/53 (67.9%)	
Group 3	37/261 (14.2%)	0/53 (0%)	
Group 4	92/261 (35.2%)	1/53 (1.9%)	
Missing	1/261 (0.4%)	1/53 (1.9%)	
Death	54/261 (20.7%)	24/53 (45.3%)	
WNT	5/72 (6.9%)	0/15 (0%)	p = 0.004***
SHH	9/59 (15.3%)	24/36 (66.7%)	

*Incidence of LCA histological variant in *TP53* wild-type and *TP53* mutant patients; **Incidence of *TP53* mutations in WNT and SHH patients; ***Incidence of death in *TP53* mutant population in WNT and SHH patients.

As previously reported, no *TP53* mutations were observed in groups 3 and 4 tumors while 17.2% (15/87) of WNT and in 37.9% (36/95) of SHH tumors harbored mutations.

**Figure 1:**
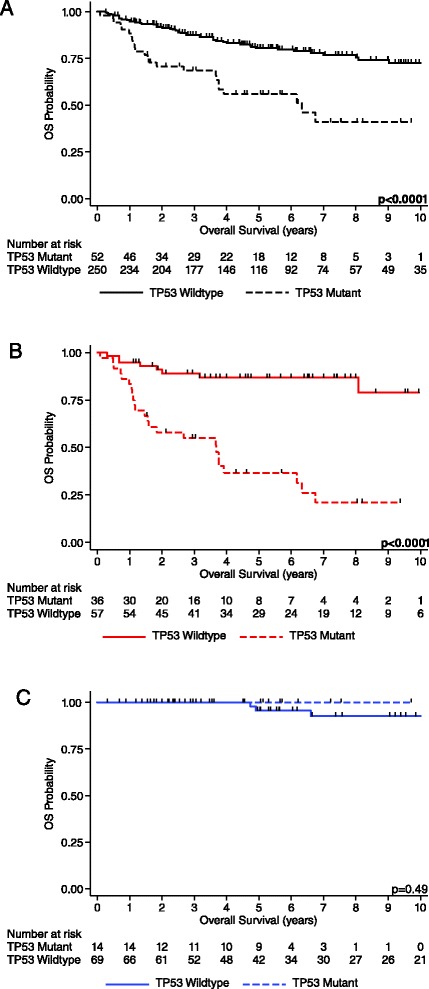
**Survival estimates for medulloblastoma patients by** ***TP53*** **status.** In all figures *TP53* status is demonstrated by solid line (wild-type) and dotted line (mutant). **A)** OS survival for all medulloblastoma patients. **B)** OS for patients with SHH medulloblastoma. **C)** OS for patients with WNT medulloblastoma.

Five-year OS for children with *TP53* wild-type and mutant tumors was 80.6% ± 2.8% and 55.9% ± 7.4% respectively (p < 0.0001) (Figure 1A). For children with SHH medulloblastoma, the 5-year OS for *TP53* wild-type patients was 86.8% ± 4.7% versus 36.6% ± 8.7% for *TP53* mutant ones (p < 0.0001) (Figure 1B). In contrast, the 5-year OS in children with WNT medulloblastoma was 95.7% ± 3% for the *TP53* wild-type and 100% for the *TP53* mutant cases (p = 0.4887) (Figure 1C)*.* The difference in patient survival between *TP53* mutant and wild-type tumors outside the WNT subgroup was more striking here than in our previous report, because the latter included patients who did not receive radiotherapy [10]. *TP53* mutations clustered with 66.7% of deaths of SHH patient in this age group. Furthermore, of the *TP53* mutant cancers that recurred, 42% (22/53) were SHH and only 16% (1/6) were WNT (p < 0.0001). Together, these findings suggest that in children with SHH tumors receiving radiotherapy, *TP53* mutation is a marker of poor outcome. This effect is entirely driven by SHH tumors, as it was not observed in WNT activated tumors. This clinical observation suggests that *TP53* mutation confer poor survival in part due to radiation resistance in non WNT tumors while this is reversed in WNT activated medulloblastomas.

*TP53* mutations confer radiation resistance in medulloblastoma cell lines. In order to determine the response to radiation in *TP53* wild-type and mutant medulloblastoma cells we used two approaches.

**Figure 2:**
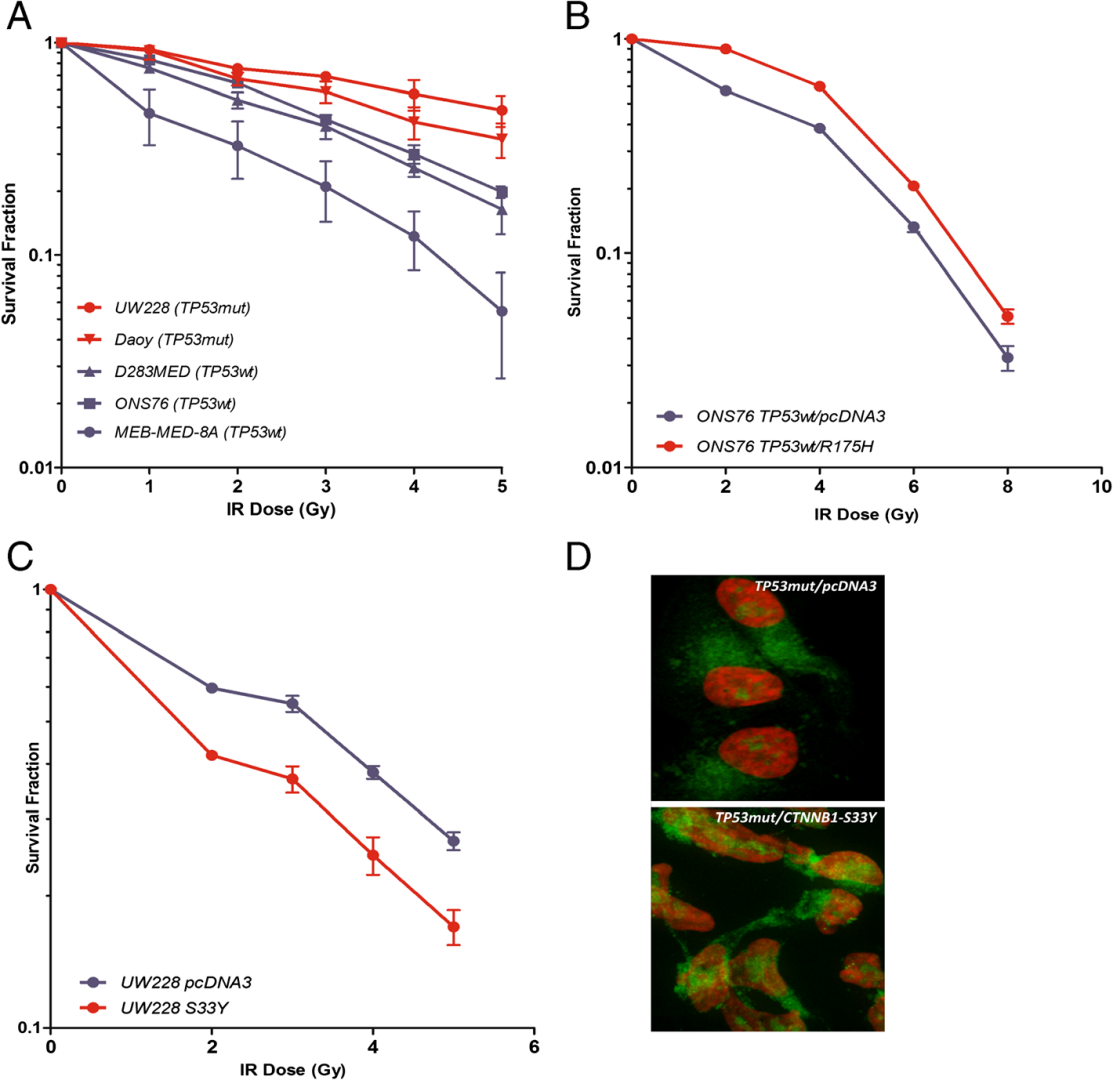
**Survival of medulloblastoma cells harboring alterations in** ***TP53*** **and** ***CTNNB1*** **after radiotherapy. A)** Clonogenic assay on a panel of *TP53* wild-type: D283MED, ONS76, MEB-MED-8A(blue) and *TP53* mutant:UW228, Daoy (red) medulloblastoma cell lines. *TP53* mutant cells exhibit superior radiation resistance (p < 0.01). **B)** Transfection of the *TP53* wild-type (blue) medulloblastoma cells (ONS76) with R175H dominant-negative *TP53* mutation (red) results in increased survival of the cells under the radiation treatment (p < 0.01). **C)** Transfection of *TP53*mut cells (UW228) (blue) with S33Y-CTNNB1 mutation (red) resulted in sensitization of cells to radiation (p < 0.05). **D)** Transfection of *TP53* mutant medulloblastoma cells (UW228) with mutant S33Y-β-catenin construct results in nuclear translocation of β-catenin (bottom panel), no nuclear translocation of β-catenin is observed in the control cells, transfected with pcDNA3 construct (top panel): β-catenin (green), nucleus (red), co-localization of β-catenin to nucleus (yellow).

First, we performed a series of clonogenic assays post radiation treatment using a panel of *TP53* wild-type and *TP53* mutant non-isogenic medulloblastoma cell lines. We observed significantly higher survival in response to radiation treatment in *TP53* mutant cell lines (Daoy and UW228), compared to *TP53* wild-type cell lines (ONS76, MEB-MED-8A and D283MED) (p < 0.01) (Figure 2A). Specifically, survival fraction at clinically relevant dose of 2Gy (SF2) was 76% ± 2.5% for UW228, 67% ± 4.7% for Daoy, 64% ± 2.2% for ONS76, 54% ± 4.7% for D283MED and 33% ± 9.9% for MEB-MED 8A cells (p < 0.001) (Additional file 1: Figure S1A).

Second, to further confirm the pro-survival effect of *TP53* mutation under radiation exposure in an isogenic system, the wild-type *TP53* cell line (ONS76) was transiently transfected with an expression vector for the dominant-negative *TP53*-R175H mutation. Expression of the mutant p53 protein resulted in a significant increase (p < 0.01) in radiation resistance in transfected cells compared to the empty-vector control (Figure 2B). The SF2 was 89% ± 2% in *TP53*-R175H transfected cells versus 57.4% ± 1.8% in the parental *TP53* wild-type cells (p < 0.001) (Additional file 1: Figure S1B).

Activation of *WNT* signaling via *CTNNB1* mutation (*S33Y*) sensitizes *TP53* mutant medulloblastoma cells to radiation. To test whether WNT pathway activation results in radiosensitivity in medulloblastoma cells, we examined the role of exon 3 mutations and subsequent *WNT* pathway activation in sensitizing *TP53* mutant medulloblastoma cells to radiation. UW228, *TP53* mutant/*CTNNB1* wild-type medulloblastoma cells were transfected with vector containing the activating S33Y mutation (*TP53*-T155N/*CTNNB1*-S33Y) and the empty vector control (*TP53*-T155N/pcDNA3). Expression of the S33Y-mutant β-catenin protein in *TP53* mutant medulloblastoma cells sensitizes these cells to radiation (p < 0.05) (Figure 2C). This was accompanied by marked nuclear translocation of β-catenin in the cells (Figure 2D)*,* similar to what has been reported for *CTNNB1*-mutant tumors [22,26,28].

These findings suggest that *TP53* mutations confer radioresistance in medulloblastoma cells while WNT activation results in radiosensitizsation.

**Figure 3:**
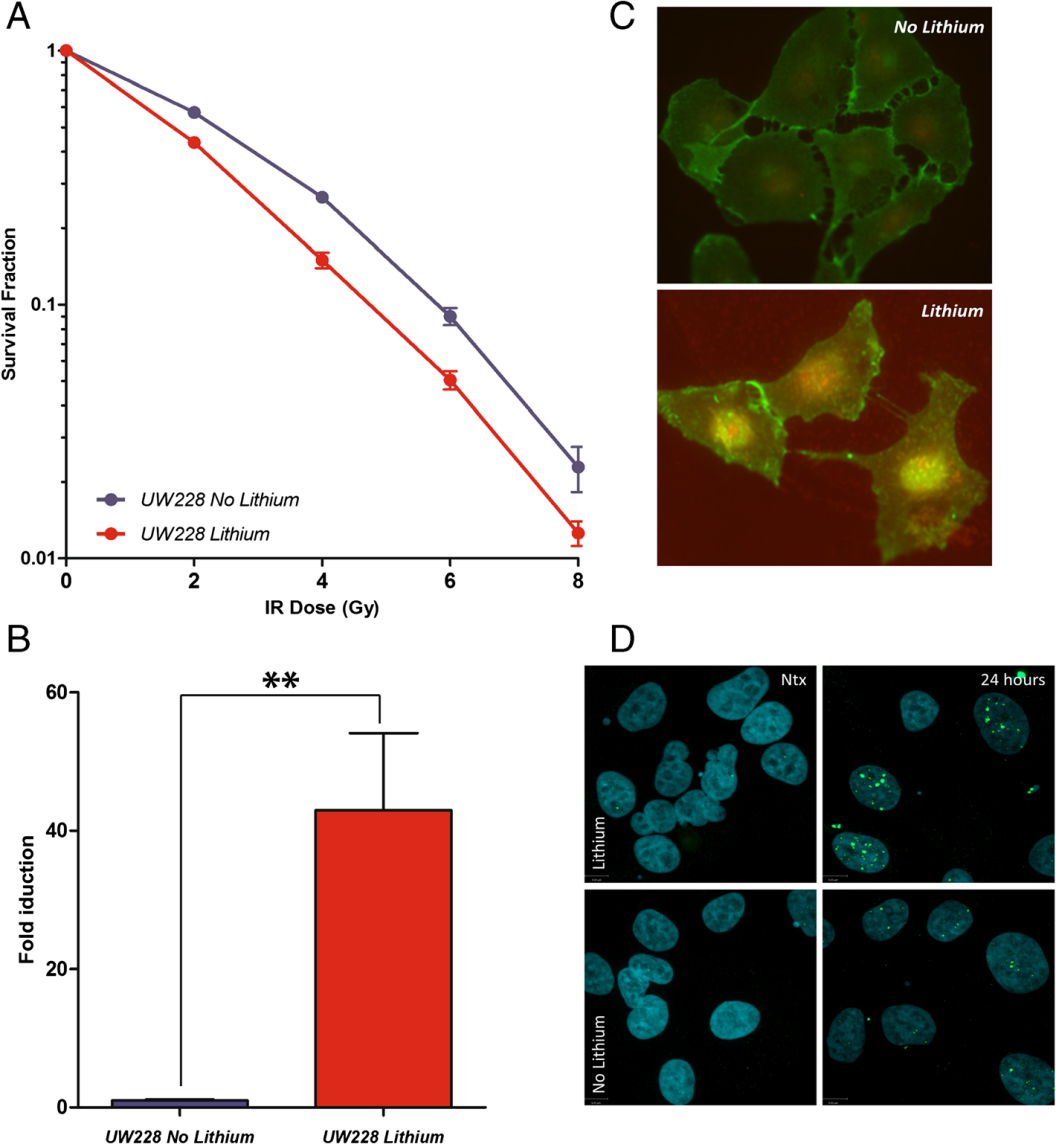
**Survival of** ***TP53*** **mutant medulloblastoma (UW228) cells after the combination treatment with 2 mM lithium and radiation. A)** Survival curves for *TP53* mutant cells given increasing radiation doses following 24 hours exposure to lithium (red) and untreated control (blue) (p < 0.01). **B)** Increase in luciferase activity in treated cells (red) over control (blue) in *TP53* mutant cells (p < 0.01). **C)** Immunofluorescent imaging of the nuclear translocation of β-catenin (bottom panel); β-catenin (green), nucleus (DAPI-red), co-localization (yellow) following 24 hours exposure to lithium. **D)** γH2AX foci in non-irradiated cells and 24 hours post irradiation: γH2AX foci (green), nucleus (blue).

Pharmacological activation of WNT by lithium reduces radioresistance in medulloblastoma with mutant *TP53*. Since lithium is known to activate *WNT* signaling via inhibition of *GSK3*β, we examined whether pharmacological intervention could mimic the radiosensitivity observed in the WNT group and sensitize *TP53* mutant cells to radiation. Pre-treatment with lithium, resulted in significantly increased (p < 0.01) radiation sensitivity of the treated cells compared to the untreated parental cells (Figure 3A) The sensitizer enhancement ratio was 1.3 at a surviving fraction of 0.10 and the SF2 was 43.5% ± 1.5% for treated cells versus 56.6% ± 3% for treated untreated cells controls (p < 0.01) (Additional file 1: Figure S1C and 1D). Activation of the *WNT* signaling pathway by lithium was demonstrated by increased luciferase activity, nuclear translocation of β-catenin in treated cells and increased number of γH2AX foci post radiation (Figure 3B,C and D). Interestingly, lithium exposure also enhances radiation sensitivity of *TP53* wild-type cells as demonstrated with ONS76 cells (Additional file 1: Figure S2A, B and C). In both *TP53* mutant and *TP53* wild-type cells we observed a higher number of γH2AX foci in lithium pre-treated cells in response to radiation treatment; at 24 hours post irradiation we found 36.6% of γH2AX foci increase in *TP53* mutant cells and 10.4% increase in *TP53* wild-type ones, suggestive of pharmacologically induced DNA break/repair mechanism impairment (Additional file 1: Figure S3).

**Figure 4:**
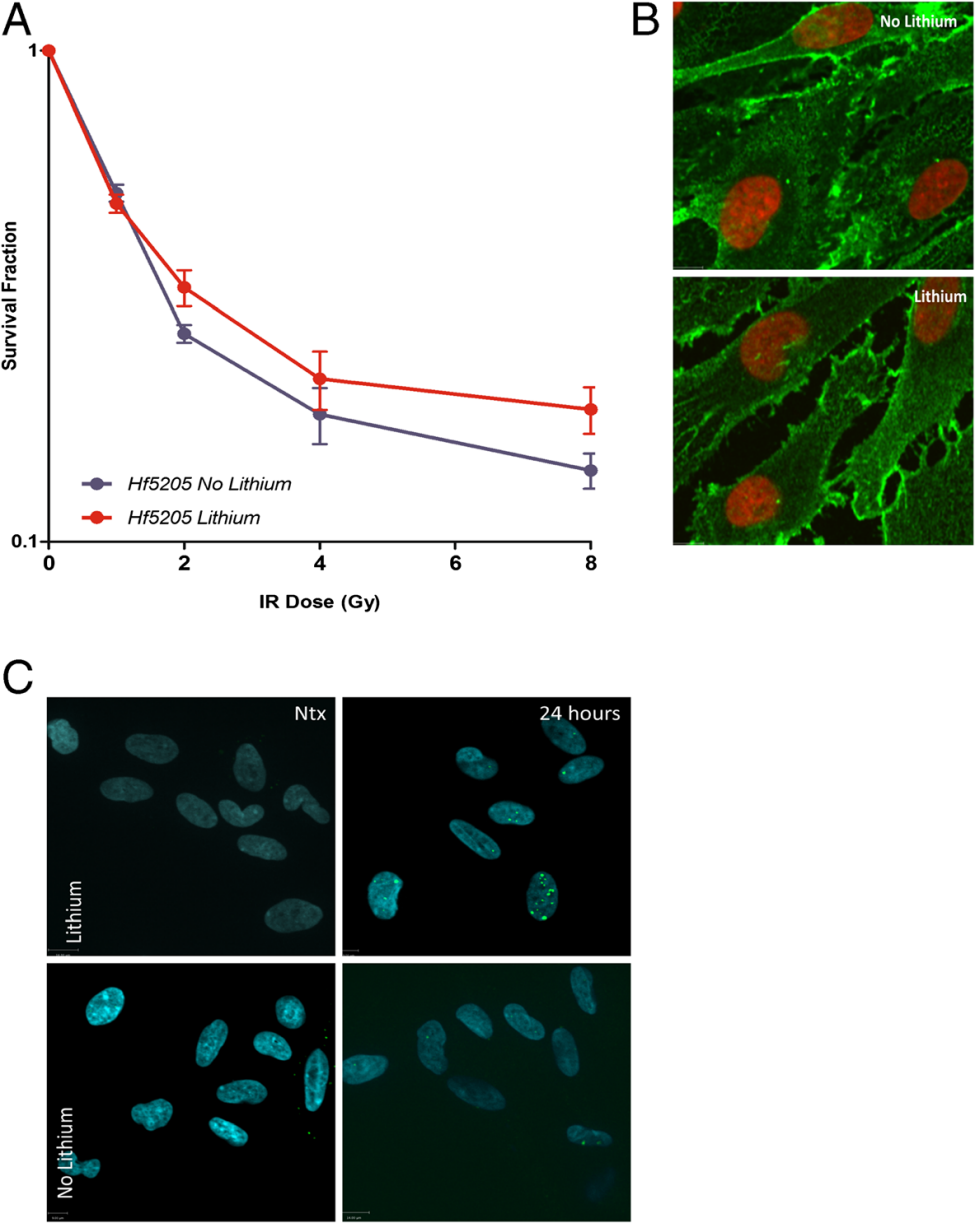
**Survival of normal neuronal stem cells (Hf5205) after the combination treatment with 2 mM lithium and radiation. A)** Survival curves for normal neuronal stem cells given increasing radiation doses following 24 hours of exposure to lithium (red) and untreated control (blue) (p = 0.054). **B)** Immunofluorescent imaging of normal neuronal stem cells; absence of the nuclear translocation of β-catenin (green) is observed in both control (top panel) and lithium treated (bottom panel) cells, nucleus (DAPI-red). **C)** γH2AX foci in non-irradiated cells and 24 hours post irradiation: γH2AX foci (green), nucleus (DAPI-blue).

Lithium does not sensitize normal neuronal stem cells to radiation. Since lithium may have detrimental effects on the developing brain if it sensitizes normal neural cells to radiation, we used Hf5205 NNSCs as a surrogate to test a combination effect of lithium and radiation therapy on normal brain cells. Pre-treatment of NNSCs with lithium did not result in decreased survival. Pre-treated cells demonstrated a slight, though not statistically significant (p = 0.15), survival advantage over their untreated counterparts (Figure 4A). SF2 analysis for both treated and untreated cells revealed a somewhat increased survival for lithium pretreated cells (33% ± 8% vs. 27% ± 3%, respectively (p = 0.05) (Additional file 1: Figure S1E). Lithium pre-treatment did not result in nuclear translocation of β-catenin (Figure 4B), nor in increased gamma γH2AX foci formation: at 24 hours post irradiation we observed 3.1 ± 0.4 foci per nucleus in lithium treated NNSCs and 2.7 ± 0.3 foci per nucleus in untreated controls (Figure 4C and Additional file 1: Figure S4).

## Discussion

In this study, we confirmed that *TP53* mutations remain a poor prognostic marker and account for a high proportion of the treatment failure in SHH subgroup medulloblastoma patients receiving radiation treatment, while *TP53* mutant WNT patients demonstrate excellent outcomes. The importance of this finding is further highlighted through molecular studies in which we demonstrated that *TP53* mutant medulloblastoma cells are more resistant to radiation. We further confirmed our results using an isogenic in vitro model, transfecting *TP53* wild-type cells with a known dominant-negative *TP53* mutation. These findings are in agreement with previously published data suggesting a context-dependent role of *TP53* in radiation response [12,19-21,44].

Activation of WNT signaling either via increased levels of β-catenin or pharmacologically with lithium radiosensitized medulloblastoma cells, abrogated the pro-survival effects of *TP53* mutations [21,45]. This effect seems to be highly tissue/tumor specific, as in the majority of tumor types increased WNT signaling is associated with tumor resistance to radiation. Recent studies suggested that WNT/β-catenin activation contributes to radiation resistance in the cancer stem cell population via the induction of chromosomal instability, deregulation of mitotic spindles and increased tolerance to DNA damage [46]. The WNT pathway has also been found to mediate radiation resistance in both mammary progenitor cells and human breast cancer cell lines [47,48], as well as in esophageal and colorectal cancers [49,50]. Furthermore, in glioblastoma multiforme mouse models and cell lines transcriptionally active β-catenin is found in the stem-like population of cells that display resistance to radiation [51].

Pharmacological radiosensitizsation of medulloblastoma cells by utilizing clinically tolerable doses of lithium prior and during radiation exposure adds a new and relatively simple dimension to the current treatment of childhood medulloblastoma. Since the decreased cell survival associated with the combination of lithium and radiation was accompanied by nuclear translocation of β-catenin and transcriptional activation of β-catenin *TCF/LEF* mediated signaling it is reasonable to suggest that this beneficial effect is due to *WNT* pathway activation in these tumors. Since lithium sensitizes all medulloblastoma cells to radiation, addition of lithium to current protocols may be beneficial to all patients but specifically to the lethal SHH *TP53* mutant subgroup. In addition, combination of lithium with small-molecule inhibitors of the *SHH* pathway may represent an attractive regimen for high risk patients with SHH medulloblastoma.

Our results are especially exciting since, in our experiments lithium does not activate *WNT* signaling in normal neuronal stem cells, nor does it sensitize them to radiation. Moreover, we were able to observe marginal improvement in NNSC survival treated with lithium, which is in agreement with published reports demonstrating protective capacity of lithium against radiation in mouse models [52,53]. Taken together, our data suggests that lithium is a tolerable and safe pharmacological agent to be added to treatment of medulloblastoma.

## Conclusion

In summary, we demonstrate here how combination of genomic and genetic analysis of brain tumors can predict differential responses to current therapies, and provide insight into ways that favorable signaling pathways may be mimicked by pharmacological intervention. We recognize that despite being safely used in humans for treatment of psychiatric disorders, lithium needs further pre-clinical evaluation prior to administration to humans for the purpose of medulloblastoma treatment. Nevertheless, our findings can also serve as proof of principle for implementation of other drugs which abrogate the detrimental effects of genetic events in the cancer therapeutic response.

## Additional file

Additional file 1: Figure S1. Survival fractions at 2Gy (SF2) and SER for medulloblastoma cells treated with radiation and combination of lithium and radiation. A) SF2 for the panel of wild-type (blue) and mutant (red) medulloblastoma cells exposed to radiation. B) SF2 for the *TP53* wild-type (blue) and R175H *TP53* mutant (red) medulloblastoma cells. C) SF2 for lithium treated *TP53* mutant cells (red) comparing to untreated control (blue). D) SER for lithium *TP53* mutant cells (red) comparing to untreated control (blue). E) SF2 for untreated NNSC (red) compared to treated with lithium (blue); *p < 0.05, **p < 0.001. Figure S2. Survival of *TP53* wild-type medulloblastoma cells after the combination treatment with lithium and radiation. A) Survival curves for *TP53* wild-type cells given increasing radiation doses following 24 hours exposure to lithium (red) and untreated control (blue) (**p < 0.01). B) Increase in luciferase activity in treated cells (red) over control (blue) in *TP53* wild-type cells (**p < 0.01). C) γH2AX foci in non-irradiated cells and 24 hours post irradiation: γH2AX foci (green), nucleus (DAPI-blue). Figure S3. DNA damage-repair response of *TP53* wild-type (ONS76) and *TP53* mutant (UW228) medulloblastoma cells to combined treatment with 2 mM lithium and radiation. Both *TP53* wild-type A) and mutant cells B) demonstrate increased number of γH2AX foci (green) in response to a combination treatment comparing to control (p < 0.0001); nucleus (DAPI-blue). Figure S4. DNA damage-repair response of normal neuronal stem cells to combined treatment with 2 mM lithium and radiation. A and B) Normal neuronal stem cells demonstrate no increase in the number of γH2AX foci (green) in response to a combination treatment comparing to control (p < 0.0001); nucleus (DAPI-blue).
