# Supplementary figures and images for: WNT activation by lithium abrogates TP53 mutation associated radiation resistance in medulloblastoma
Source: Acta Neuropathol Commun. 2014 Dec 24;2:174. doi: 10.1186/s40478-014-0174-y (PMC4297452; doi:10.1186/s40478-014-0174-y)

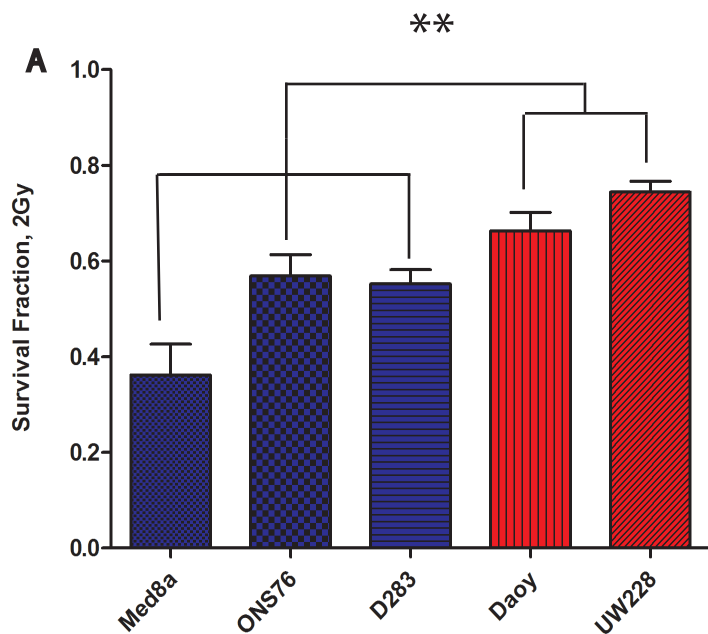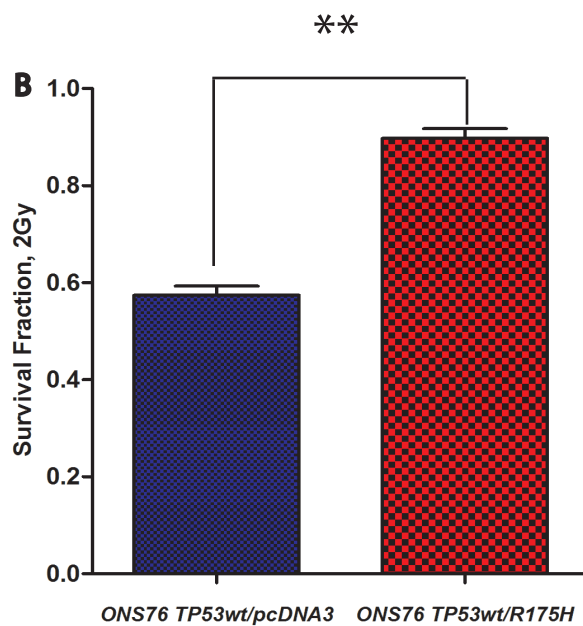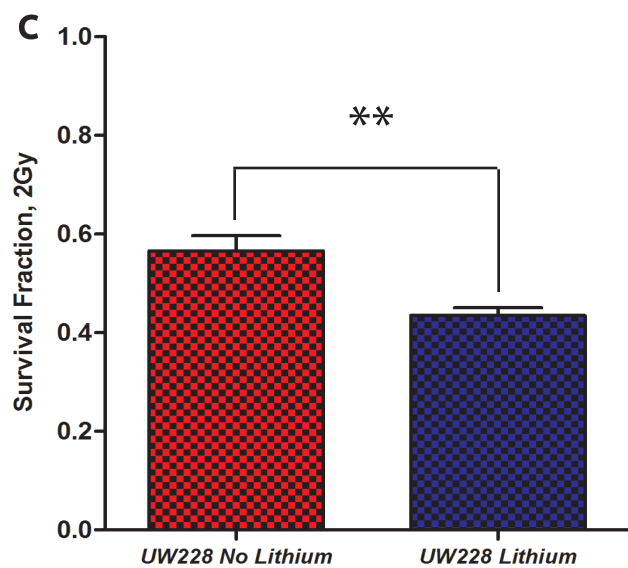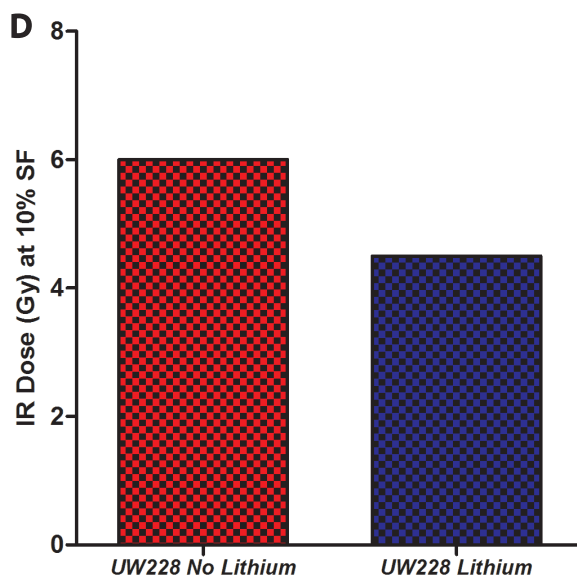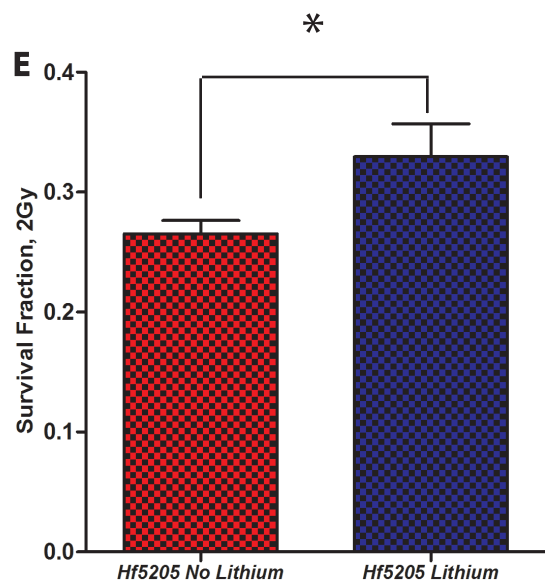

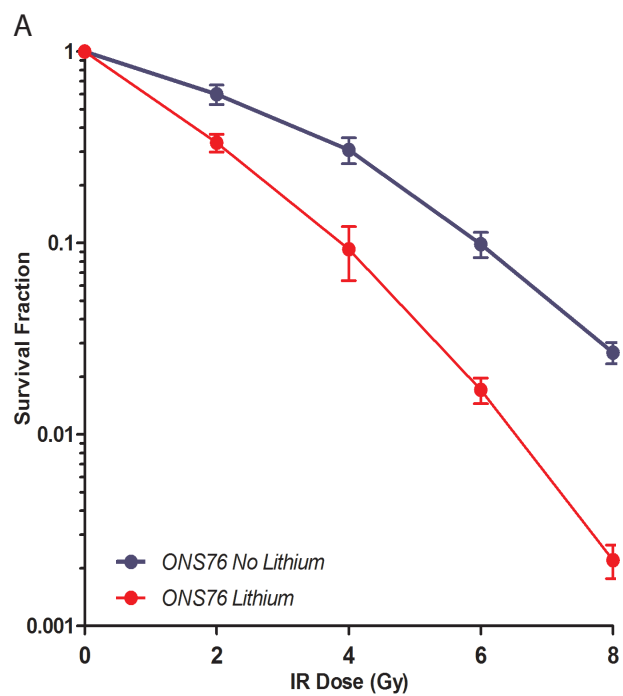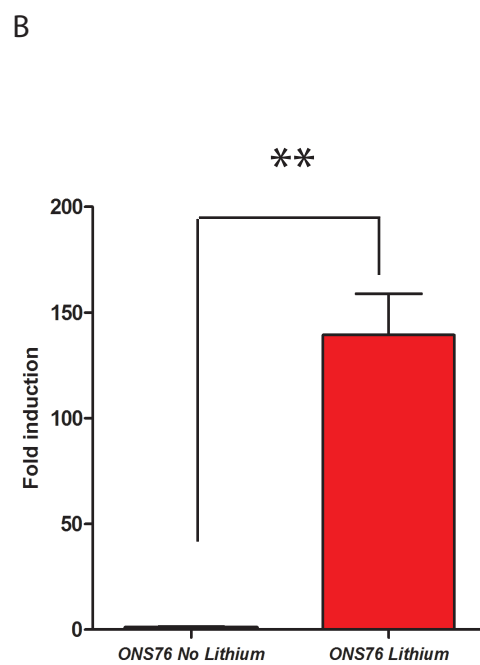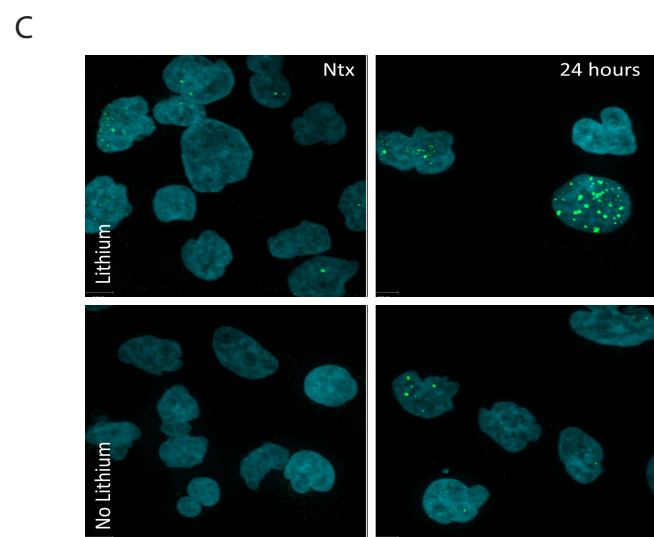

A

*TP53 mutant cells*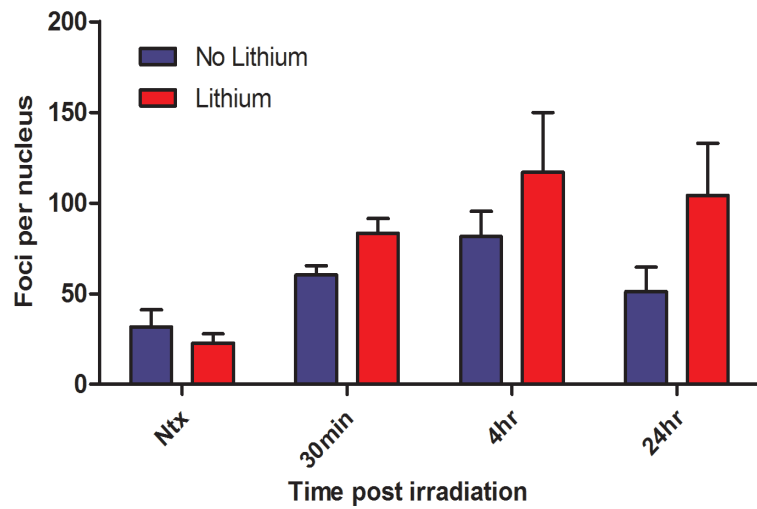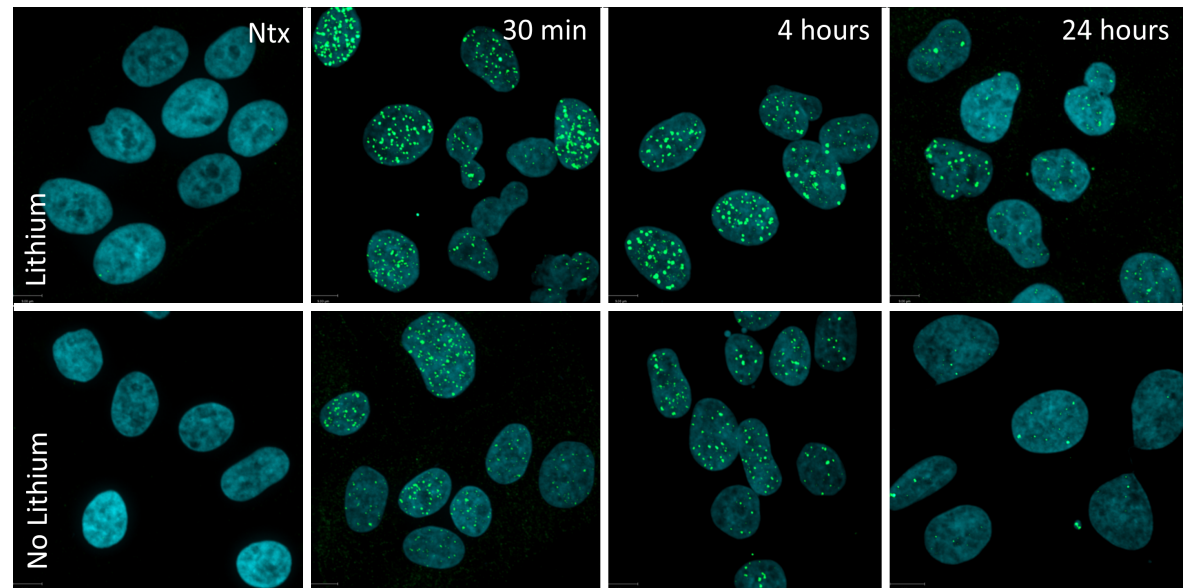

B

*TP53 wild-type cells*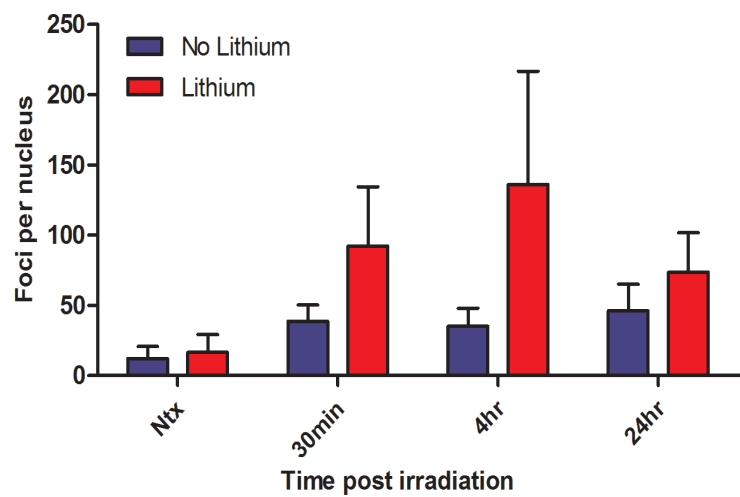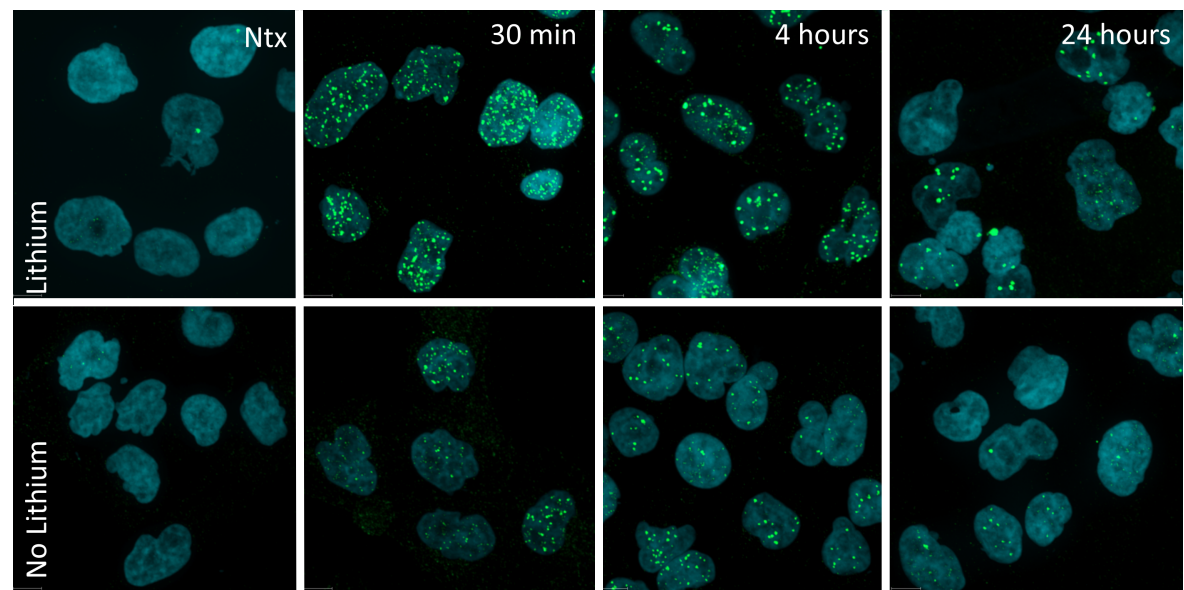

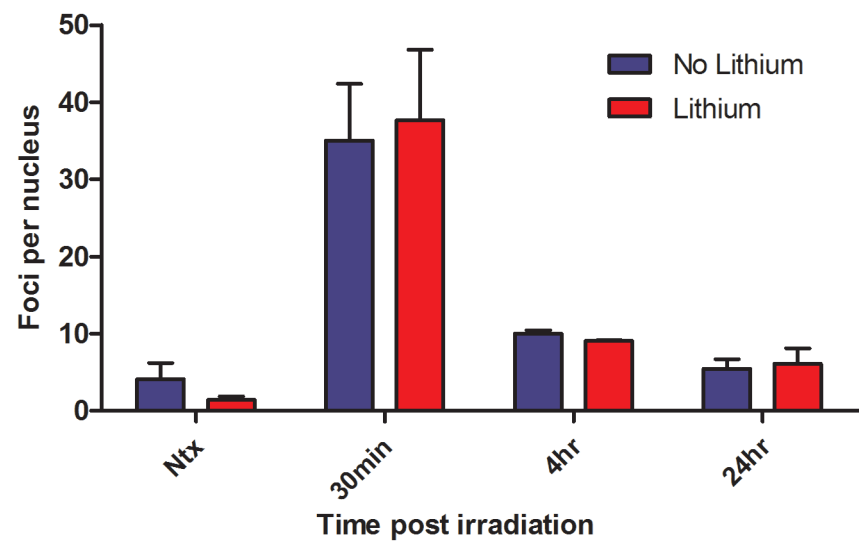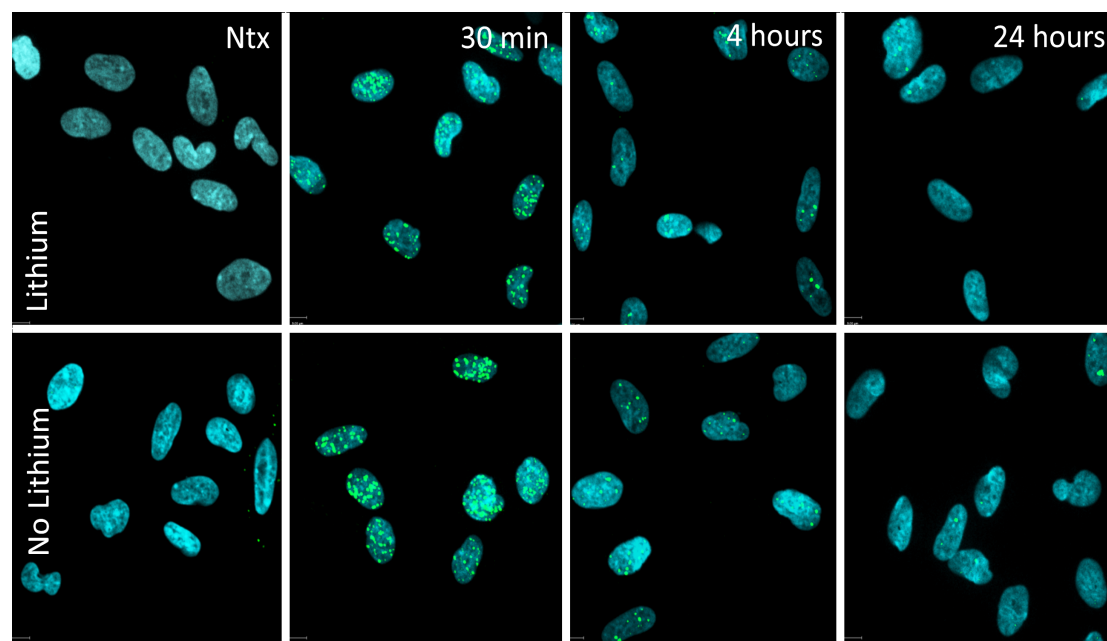

Supplement: Additional file 1: Figure S1. — Survival fractions at 2Gy (SF2) and SER for medulloblastoma cells treated with radiation and combination of lithium and radiation. A) SF2 for the panel of wild-type (blue) and mutant (red) medulloblastoma cells exposed to radiation. B) SF2 for the TP53 wild-type (blue) and R175H TP53 mutant (red) medulloblastoma cells. C) SF2 for lithium treated TP53 mutant cells (red) comparing to untreated control (blue). D) SER for lithium TP53 mutant cells (red) comparing to untreated control (blue). E) SF2 for untreated NNSC (red) compared to treated with lithium (blue); *p < 0.05, **p < 0.001. Figure S2. Survival of TP53 wild-type medulloblastoma cells after the combination treatment with lithium and radiation. A) Survival curves for TP53 wild-type cells given increasing radiation doses following 24 hours exposure to lithium (red) and untreated control (blue) (**p < 0.01). B) Increase in luciferase activity in treated cells (red) over control (blue) in TP53 wild-type cells (**p < 0.01). C) γH2AX foci in non-irradiated cells and 24 hours post irradiation: γH2AX foci (green), nucleus (DAPI-blue). Figure S3. DNA damage-repair response of TP53 wild-type (ONS76) and TP53 mutant (UW228) medulloblastoma cells to combined treatment with 2 mM lithium and radiation. Both TP53 wild-type A) and mutant cells B) demonstrate increased number of γH2AX foci (green) in response to a combination treatment comparing to control (p < 0.0001); nucleus (DAPI-blue). Figure S4. DNA damage-repair response of normal neuronal stem cells to combined treatment with 2 mM lithium and radiation. A and B) Normal neuronal stem cells demonstrate no increase in the number of γH2AX foci (green) in response to a combination treatment comparing to control (p < 0.0001); nucleus (DAPI-blue). [file 40478_2014_174_MOESM1_ESM.pdf]
